# Roles of CDK and DDK in Genome Duplication and Maintenance: Meiotic Singularities

**DOI:** 10.3390/genes8030105

**Published:** 2017-03-20

**Authors:** Blanca Gómez-Escoda, Pei-Yun Jenny Wu

**Affiliations:** Genome Duplication and Maintenance Team, Institute of Genetics and Development of Rennes, CNRS UMR 6290, 2 av. du Pr. Léon Bernard, 35043 Rennes, France; blanca.gomez.escoda@gmail.com

**Keywords:** cyclin-dependent kinase, Dbf4-dependent kinase, mitosis, meiosis, genome duplication, meiotic recombination, quantitative model

## Abstract

Cells reproduce using two types of divisions: mitosis, which generates two daughter cells each with the same genomic content as the mother cell, and meiosis, which reduces the number of chromosomes of the parent cell by half and gives rise to four gametes. The mechanisms that promote the proper progression of the mitotic and meiotic cycles are highly conserved and controlled. They require the activities of two types of serine-threonine kinases, the cyclin-dependent kinases (CDKs) and the Dbf4-dependent kinase (DDK). CDK and DDK are essential for genome duplication and maintenance in both mitotic and meiotic divisions. In this review, we aim to highlight how these kinases cooperate to orchestrate diverse processes during cellular reproduction, focusing on meiosis-specific adaptions of their regulation and functions in DNA metabolism.

## 1. Introduction

The ability to reproduce is a defining criterion for all living organisms. In vegetatively growing cells, this is achieved through mitotic divisions, which give rise to two daughter cells with equal genomic contents. When cells engage in sexual reproduction, they undergo meiosis: diploid cells produce four haploid gametes, each containing half of the genetic content of the mother cells. Meiosis is a specialized reductional division in which a single genome duplication is followed by two consecutive rounds of chromosome segregation (referred to as meiosis I and II). One key outcome of meiosis is the generation of increased genetic diversity in the gametes through recombination, a central feature of sexual reproduction [1,2]. Although mitosis and meiosis share a number of events, including DNA replication and chromosome segregation, there are critical differences in the regulation and execution of these processes.

The mechanisms that drive both mitosis and meiosis are tightly controlled, and this relies on the functions of two conserved types of serine-threonine kinases, the cyclin-dependent kinases (CDK) and the Dbf4-dependent kinase (DDK) (reviewed in [3,4]). In a mitotic cycle, CDK activity regulates cell cycle progression, with essential roles at its major transitions: G1/S (DNA replication) and G2/M (chromosome segregation) [5]. Moreover, CDK modulates multiple cellular processes including metabolism, transcription, differentiation, and DNA repair (reviewed in [6,7]). Similarly, DDK is a critical regulator of DNA replication, chromosome segregation, centromeric heterochromatin formation, and genome maintenance [3,8,9,10,11,12]. Beyond these functions in proliferating cells, both kinases also possess meiosis-specific roles, such as in meiotic recombination and chromosome segregation [13,14,15,16,17,18]. In many of these pathways, consensus phosphorylation sites for both CDK and DDK have been identified in common target substrates [18,19,20,21,22], and studies have shown an important interplay between these kinases in distinct mitotic and meiotic processes.

**Figure 1:**
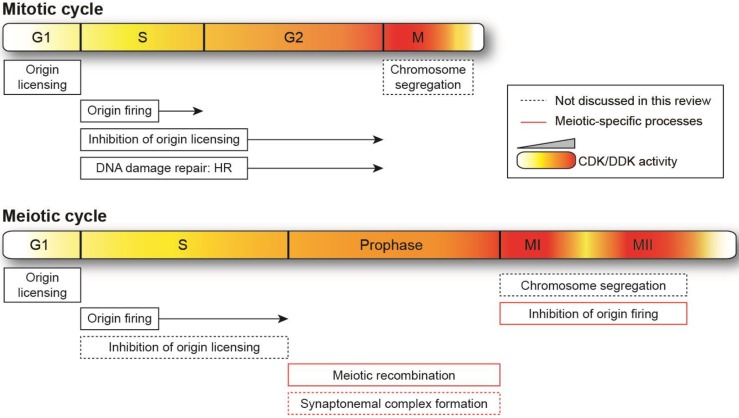
Schematic representation of mitotic and meiotic cycles. Relative changes in cyclin-dependent kinase (CDK) and Dbf4-dependent kinase (DDK) activity are illustrated by the intensity of the gradient (yellow-red), with more intense red denoting higher levels. For ease of visualization, the higher activities required in meiosis vs. mitosis are not depicted. The lengths of the cell cycle phases are not shown to scale. HR: homologous recombination. Meiosis-specific events are highlighted in red boxes, and processes that are not discussed in this review are indicated by dotted lines.

In this review, we will discuss the regulation and requirements for CDK and DDK in the mitotic and meiotic cycles, in particular in the events surrounding genome duplication and maintenance (Figure 1). As a number of reviews have addressed the activities of these kinases in proliferating cells [3,4,23], we will pay special attention to the modification of their roles during sexual reproduction. First, we will present evidence for quantitative models for how CDK and DDK activities ensure the temporal progression of mitotic and meiotic events. We will then introduce additional features of their control that are specific to meiosis. Next, we will consider the functions of CDK and DDK in genome duplication and the prevention of re-replication by high CDK activity. Finally, we will focus on the mechanisms by which these kinases coordinate DNA replication with the formation of programmed DNA double-strand breaks (DSBs) and their repair during meiosis. While this review will not exhaustively cover all CDK and DDK functions, we aim to highlight how these two kinases regulate diverse processes that are essential to cellular reproduction.

## 2. Regulation of the Mitotic Cycle by Quantitative Changes in Cyclin-Dependent Kinase and Dbf4-Dependent Kinase Activities 

Active CDK and DDK are comprised of two components: a catalytic subunit and a regulatory protein required for kinase activation. In the case of CDK, one kinase can associate with diverse cyclins in a cell cycle-regulated manner ([5] and discussed in further detail below). The regulation of DDK is perhaps more straightforward since its function is modulated by one major partner, Dbf4, and by a second related protein that is found only in vertebrates, Drf1 [24,25,26,27]. Control of both CDK and DDK activities is integral to their roles in driving the mitotic and meiotic cycles.

In eukaryotes, cell cycle transitions are brought about by dynamic interactions between cyclins and CDKs. Multiple cyclin-CDK pairs have been identified in mammalian cells, and different combinations act at distinct stages of the cell cycle: for instance, cyclin D-Cdk4/6 in G1, cyclin E-Cdk2 at the G1/S transition, cyclin A-Cdk2 in S, and cyclin B-Cdk1 at the G2/M transition [5]. Even in simpler systems such as the unicellular budding yeast *Saccharomyces cerevisiae*, there are nine cyclins (Clns 1–3 and Clbs 1–6) that activate the single cell cycle CDK, Cdc28 [5]. Cyclins display different profiles of expression and degradation, and dedicated cyclin-CDK complexes are believed to generate the substrate specificities that promote particular cell cycle transitions and ensure the order of cell cycle events. However, it has become clear that cell cycle progression per se does not require diversity in cyclin and CDK interactions but is rather directly driven by CDK activity levels. This quantitative model of the cell cycle proposes that S phase and mitosis rely on low and high CDK activity thresholds, respectively, and that no qualitatively different cyclin-CDK complexes are necessary [28,29]. A large body of evidence from a variety of organisms has now provided support for this model. First, there is a clear redundancy in cyclin function. For instance, in budding yeast mutants lacking the S phase cyclins Clb5 and Clb6, the Clb1–4 mitotic cyclins allow DNA replication that is delayed but nevertheless involves both early and late firing replication origins [30]. In addition, when expressed under the control of the *CLB5* promoter, Clb2 cyclin alone, in the absence of all other Clbs, is able to perform both S phase and mitotic functions [31]. Similar observations have been made in the fission yeast *Schizosaccharomyces pombe.* Indeed, while cell cycle regulation is orchestrated by four cyclins (Cig1, Cig2, Puc1 for G1/S and Cdc13 for mitosis) and one CDK (Cdc2), the Cdc13-Cdc2 complex is sufficient to sustain cell cycle progression when all other cyclin genes are deleted [28]. This redundancy is not unique to simple eukaryotes and extends to more complex systems. One example is provided by work in *Xenopus* egg extracts, where relocalizing the mitotic cyclin B1-Cdk1 complex from the cytoplasm to the nucleus reveals its ability to promote S phase [32]. Furthermore, mouse embryonic fibroblasts lacking all three D-type cyclins that normally function in early G1 are able to proliferate, and the triple knockout mice are viable until E16.5 [33]. Next, CDKs have also been demonstrated to be redundant in function. Remarkably, in the mouse embryo, the mitotic CDK Cdk1 itself supports cell proliferation in the absence of all interphase CDKs (Cdk2, Cdk3, Cdk4 and Cdk6) until midgestation [34]. Finally, direct evidence for a quantitative model that governs the control of cell proliferation was provided by work in the fission yeast. In this organism, oscillations in CDK activity generated by chemical inhibition of a fusion protein consisting of Cdc13 (cyclin B) and Cdc2 (CDK) are sufficient to autonomously trigger passage through the cell cycle, even when the level of the Cdc13-Cdc2 protein remains constant [35]. Strikingly, regardless of the cell cycle phase that they are in, cells respond directly to the CDK levels that are imposed. For example, cells in G1 that experience high levels of CDK activity will proceed immediately into mitosis while at the same time duplicating their genomes [35]; this is consistent with previous data showing that the fusion of human mitotic cells with G1 or G2 cells induces interphase chromatin to undergo condensation [36]. This direct response is also observed at the level of gene expression, where the periodic transcription of cell cycle gene clusters is controlled by CDK activity [37,38]. Complementary to these findings, recent work suggests that the differential phosphorylation of CDK targets may be due to their distinct sensitivities to CDK activity, as early (G1/S) substrates are modified at lower activity levels than late (G2/M) substrates [39]. Collectively, these results demonstrate that oscillations in CDK activity, rather than the specificities of individual cyclin-CDK complexes, drive the timing and directionality of the events in the mitotic cycle.

In contrast to the requirement for CDK, much less is known about the profile of DDK activity, despite its key functions in distinct steps of the cell cycle. Nevertheless, an analogy may be made to the quantitative model for CDK. In mitotic cycles, the DDK (Cdc7 in most organisms, Hsk1 in the fission yeast) is activated by association with a cyclin-like regulatory subunit, Dbf4. Although a second regulator, Drf1, has been identified in vertebrates [24,25,26,27], this review will focus on Dbf4-DDK complexes. During vegetative growth, a peak of DDK activity occurs during S phase due to the oscillation in Dbf4 protein levels, which are low in G1, increased at the G1/S transition, maintained high during S phase, and reduced during G2/M [20,40,41,42,43]. The levels of the Cdc7 kinase itself, however, remain constant throughout the cell cycle [40,44]. This profile of kinase activity is consistent with the role of DDK in S phase entry, where it is limiting for replication initiation [45,46,47]. Therefore, quantitative regulation may be a unifying principle for the essential enzymes that control the different critical events during the mitotic cycle. 

## 3. A Quantitative Model for Cyclin-Dependent Kinase and Dbf4-Dependent Kinase Activities in Meiosis

Given the similarities between mitosis and meiosis, could the quantitative model for CDK activity also apply to meiotic progression? Initial studies in the budding yeast suggested that there may be a more specific prerequisite for cyclin-CDK complexes during meiosis. First, the major mitotic cyclin Clb2 is not significantly expressed in meiosis [48,49], while Clbs 1, 3, and 4 contribute to entry into meiosis I and are essential for the progression from meiosis I to meiosis II [50,51]. In addition, although the functions of Clb5 and Clb6 in the control of S phase during a vegetative cycle can be replaced by other cyclins [30,52], they are indispensable for the initiation of pre-meiotic S phase [53,54,55]. Interestingly, the role of Clb5 can be bypassed by fusing Clb3 with the Clb5 hydrophobic patch that influences substrate interactions [55], hinting at a specific meiotic function for this domain. The importance of regulation by distinct cyclin-CDK pairs appears to extend to processes that only occur in meiosis. For instance, the initiation of meiotic recombination is defective in the absence of Clb5 and Clb6 [56]. Similarly, in the fission yeast, the lack of either the meiotic cyclin Rem1 or the G1/S cyclins Cig1, Cig2, and Puc1 reduces intergenic recombination and spore viability [57,58]. These requirements are also found in the mouse, where cyclin E1/E2 defective males show a normal cell cycle but have defects in spermatogenesis [59]. Moreover, the lack of cyclin A1 blocks this process before the first meiotic division, indicating that its functions cannot be complemented by the B type cyclins that are present in the cells [60]. Finally, the control of CDK activity provided by multiple cyclin-CDK complexes during meiosis is further complicated by the existence of additional regulators in systems such as the budding yeast, where the Ime2 meiosis-specific serine-threonine kinase is required for pre-meiotic S phase and for the meiotic divisions [53,54,61,62]. Ime2 has both sequence and functional homology with human CDK2 [63], and some of its key substrates are also targets of CDK/Cdc28 [64]; it thus acts as a companion kinase to CDK in this process. All together, these findings suggest that diversity in CDK and CDK-related activities are essential for cells to progress through meiosis.

However, recent studies in the fission yeast have indicated that the quantitative model may also apply to the succession of meiotic events. In this organism, in addition to the four cyclins that participate in mitotic cycles, there are two meiosis-specific cyclins (Rem1 and Crs1) [58,65]. Cig2, Rem1, and Crs1 have been shown to partner with CDK in pre-meiotic S phase [58,65,66]. Removal of cyclin genes shows additive effects, with multiple deletions displaying greater delays in replication initiation compared to single mutants [57]. The single Cdc13-Cdc2 fusion protein mentioned above [35] was then tested for its ability to drive meiotic progression in the absence of other cyclin-CDKs [57]. Interestingly, while Cdc13-Cdc2 permits relatively efficient completion of pre-meiotic S phase, cells almost completely fail to undergo meiotic divisions. Strikingly, four copies of this active CDK module allow cells to proceed through meiosis [57]. These results imply that a variety of qualitatively different complexes is not required for meiotic progression and that a higher level of CDK activity is necessary for meiosis, in particular for later meiotic events. This increased sensitivity of post-replication processes to CDK activity levels was previously observed in the budding yeast using a chemically modulatable form of CDK (Cdc28-as1), as blocking pre-meiotic DNA replication required 10 times more inhibitor than preventing meiotic divisions and spore formation [61]. Thus, rather than a need for multiple cyclins, the diversity in cyclin-CDK complexes may simply give rise to a cumulatively higher level of CDK activity for meiosis. Although evidence for a quantitative model for meiotic CDK activity has so far only been provided in yeast, it is interesting to speculate that in meiosis as in mitosis, specific cyclin-CDK interactions have an additive effect and that it is the changes in CDK activity that are critical for driving these cycles.

Similarly, DDK is required for a succession of meiotic events, from replication initiation to double-strand break formation to the commitment to reductional chromosome segregation during meiosis I [16,18]. Interestingly, its activity increases as cells progress through S phase to later steps. As is the case for CDK, lower levels are necessary for origin firing than for DSB formation [14,16,67,68]. Interestingly, an additional layer of regulation is provided by the DDK-like protein Spo4 in the fission yeast, perhaps in a manner analogous to the Ime2 CDK-related kinase. Spo4 and its regulator Spo6 are expressed exclusively in meiosis, and while Spo4 is dispensable for meiotic replication, it contributes to meiotic chromosome segregation [69]. Consistent with this, its absence only affects late events and results in abnormally elongated anaphase II spindles that abolish the linear order of nuclei in the ascus [70]. These observations suggest that higher levels of DDK and related kinase activities are important for the execution of meiotic recombination and chromosome segregation.

Therefore, although the requirements for CDK and DDK during meiosis are more complex than for the mitotic cycle, their functions may both operate through the regulation of their overall activities. Low thresholds are sufficient for initiating pre-meiotic S phase, while higher levels are necessary for later events. However, it is possible that a more subtle regulation of CDK and DDK is required in meiosis as both kinases coordinate genome duplication with other functions (see below). Indeed, one particularity of meiosis is the passage from meiosis I to II, during which chromosome segregation is followed by a second round of division without an intervening S phase. At this step, CDK activity must be sufficiently low to ensure chromosome segregation but high enough to block replication and progress into meiosis II (this will be addressed in a later section). In contrast to the mitotic cycle, these complexities may involve the implementation of additional thresholds for the different processes that are specific to meiosis. This may underlie the apparent necessity for the qualitatively different activities described above. Thus, regardless of the mechanistic details of these controls, it has become clear that the dynamics of CDK and DDK activities play critical roles in ensuring meiotic progression.

## 4. Further Specificities of Cyclin-Dependent Kinase and Dbf4-Dependent Kinase Regulation in Meiotic Cycles

The regulation of CDK and DDK is fundamental to both mitotic and meiotic progression. Interestingly, although these kinases control some of the same events in these distinct cell cycles, there are clear differences in how their activities are modulated. For CDK, binding to diverse cyclins is a key part of kinase regulation, and this may provide quantitative inputs rather than qualitatively distinct functions, as discussed above. Moreover, there are additional mechanisms that contribute to meiosis-specific changes in CDK activity. For instance, the essential CDK activating kinase (CAK) constitutively simulates CDK [71,72], and further activation then occurs through CDK-dependent phosphorylation followed by targeted degradation of the CDK inhibitor (CKI) [73,74]. This is illustrated in the mitotic cycle in the budding yeast, where the G1 Cln-Cdc28 complexes phosphorylate the CKI Sic1 to allow Clb-Cdc28 activation for triggering S phase onset [74]. In contrast, regulation of pre-meiotic S phase entry is brought about by a different process. Indeed, Sic1 proteolysis in meiosis does not require Cdc28 but rather relies on the Ime2 CDK-like kinase, which is activated by Cak1 [75]. Ime2, therefore, has a crucial role in decreasing the levels of Sic1, thus bringing about the activation of the CDK [53,61]. Furthermore, CAK is transcriptionally and post-translationally regulated during meiosis, whereas its levels remain constant during the mitotic cycle [72,75]. These differences between the regulation of CDK during mitosis and meiosis highlight the singularities in these cycles.

Similarly, the control of DDK activity during meiosis also involves supplementary layers of regulation. As mentioned above, DDK modulation in proliferating cells occurs through alteration in the levels of its regulatory subunit, which peaks in S phase [40,41,42], while the DDK itself is present at constant levels [40,44]. In contrast, during meiosis in the budding yeast, DDK/CDC*7* transcript levels are increased throughout meiotic progression, being low in S phase and rising to reach a maximum around the onset of recombination [44]. As DDK activity is limiting in particular for later meiotic events, it is tempting to speculate that this additional mechanism may contribute to the temporal ordering of meiotic stages.

The differential and more complex regulation of CDK and DDK in meiosis vs. mitosis suggests that a fine-tuned, meiosis-specific activation of these kinases may be important to ensure proper meiotic progression. Together with the higher levels of CDK and DDK activities that are crucial for later meiotic steps, these additional controls may participate in orchestrating the program of meiosis.

## 5. Genome Duplication in Mitosis and Meiosis

Genome duplication is an essential step during both vegetative cell growth and sexual differentiation. Although equivalent replication machineries are required for mitotic and pre-meiotic S phases [76,77,78], a number of differences have been reported for genome duplication between these two cycles. In all systems studied to date, pre-meiotic S phase is longer than mitotic S phase [79,80]. Strikingly, this does not occur as a result of activating distinct sets of origins in the genome [81,82,83]. Instead, as demonstrated by work in the fission yeast, both the duration of S phase and the pre-meiotic replication program are dependent on the environmental conditions rather than commitment to meiosis per se: inducing meiosis after temporary nitrogen deprivation results in an identical origin usage profile and length of S phase as in cells that enter a mitotic cycle in the same conditions [83]. Interestingly, the extended length of genome duplication in meiosis has been proposed to allow for a coordination of replication with concomitant processes [80,84], such as the formation of DSBs for meiotic recombination. However, experimentally shortening S phase does not affect the ability of fission yeast cells to generate DSBs [83], suggesting that the duration of this critical step may be important for other meiosis-specific functions. Nevertheless, pre-meiotic DNA replication is tightly coupled to meiotic recombination, and this critical coordination will be discussed in a later section.

DNA replication in both mitotic and meiotic cycles is regulated by CDK and DDK, which phosphorylate multiple, evolutionary conserved substrates [21,22,53,54,85,86,87]. Many of these proteins are targets of both kinases, and CDK phosphorylation has been shown to prime certain substrates for DDK. For instance, phosphorylation of subunits of the Mcm helicase by CDKs facilitates DDK/Cdc7-dependent modification of Mcm2, revealing a collaboration between these two kinases for entry into S phase [19]. Consistent with this observation, initial studies in the budding yeast suggest that DDK performs its functions for replication only when S phase CDK (S-CDK) is also active or has been previously active [20]. In contrast, in vitro analyses using purified proteins and *S. cerevisiae* extracts show that DDK drives recruitment of the Cdc45 replication initiation factor to origins before S-CDK activation [88]. More recently, assays using a fully reconstituted replication initiation system from the budding yeast demonstrate that DDK can act either before or after CDK to phosphorylate Mcm and that the order in which the kinases function does not affect replication efficiency [89]. These different conclusions indicate that there may not be a defined order of action for CDK and DDK in the activation of origin firing or that particular temporal requirements may be linked to specific conditions. Regardless, it is clear that the cooperation between the two kinases is essential for genome duplication. As the individual functions of CDK and DDK during replication initiation in proliferating cells have been the subject of excellent reviews (for example, see [3,4]), we will focus on aspects that are specific to the meiotic cycle.

During the passage from meiosis I to II, genome duplication must be prevented for the generation of viable haploid gametes. Importantly, CDK has a dual role in activating replication as well as inhibiting re-initiation through blocking replication factor assembly at fired origins (reviewed in [90]). Therefore, while CDK activity must decrease to allow chromosome segregation, it has to remain sufficiently high to block replication and favor progression into meiosis II. In starfish oocytes, this is brought about by newly assembled cyclin B-Cdc2 complexes that suppress DNA replication between the two meiotic divisions [91]. The maintenance of adequate CDK activity can also be achieved by downregulation of the CDK-inhibiting kinase Wee1 in meiosis I, as shown in *Xenopus* oocytes [92,93]. Following the same logic but an alternative process, meiosis-specific modulation of the anaphase promoting complex (APC) results in incomplete degradation of cyclin B after meiosis I in a number of systems (reviewed in [94,95]). Finally, additional parallel pathways have been demonstrated to participate in this regulation: after the completion of meiosis I in *Xenopus* oocytes, re-activation of cyclin B-Cdc2 by the Mos kinase is critical for preventing an additional round of genome duplication prior to meiosis II [96,97]. The molecular mechanisms that are responsible for blocking DNA synthesis are similar to those used in mitotic cycles, where CDK activity rises during S phase and inhibits origin re-licensing through inhibitory phosphorylation of different pre-replicative complex components (reviewed in [90]). For instance, in the fission yeast, subunits of the Mcm helicase are no longer bound to chromatin between meiosis I and II [77], and a reduction in CDK activity during this transition increases DNA replication, most likely by increasing the efficiency of Mcm2–7 chromatin loading [98]. Taken together, these studies provide evidence that CDK regulation of re-replication is essential not only for the faithful duplication of the genomic material during the mitotic cycles but also for a successful outcome to meiosis. In contrast, while DDK does not have a direct role in ensuring that the genome is duplicated only once per cell division cycle, inhibition of its function is triggered by pathways that prevent re-replication. Studies in the budding yeast suggest that Dbf4 degradation, which begins at the metaphase to anaphase transition, may ensure that replication complexes that are assembled as cells exit mitosis are unable to fire prior to S phase [41,43]. In proliferating mammalian cells, phosphorylation of DDK/Cdc7 by CDK1 in prometaphase results in loss of Cdc7 from chromatin and specifically from origins, thus preventing inappropriate re-initiation [99]. Interestingly, an analogous phenomenon is observed in *Xenopus* oocytes between meiosis I and II, where the normally nuclear Cdc7 protein is translocated into the cytoplasm, perhaps as an extra layer of control to ensure replication inhibition at this stage [100]. Therefore, the pathways that limit DNA replication during a mitotic cycle are also relevant for meiosis. It is thus clear that both CDK and DDK are indispensable for preserving the singularity of meiosis, in which two nuclear divisions are preceded by a single genome duplication.

## 6. Coordination between Pre-Meiotic Replication and DNA Double-Strand Break Formation

A defining feature of sexual reproduction is the generation of increased genetic diversity through meiotic recombination. While DSBs occur during mitotic cycles as a consequence of endogenous and exogenous challenges, meiotic DSBs are induced by a highly regulated mechanism that follows pre-meiotic DNA replication [101]. Indeed, DSB formation in meiosis is catalyzed by the conserved Spo11 enzyme and is restricted to a time interval between replication and chromosome segregation. This is important for both (1) their role in the establishment of physical links between homologous chromosomes that are crucial for accurate segregation in meiosis I and (2) their subsequent recombination and repair. Although complete duplication of the genome is not a prerequisite for the generation of DSBs in the budding and fission yeasts [76,82,102,103,104], a clear connection has been established between these processes. In the budding yeast, inducing a delay in the timing of duplication of a genomic region results in a corresponding delay in local DSB formation [105,106]. Moreover, the profile of replication initiation along the chromosomes has been demonstrated to be a major determinant in the frequencies and genome-wide distribution of DSB formation in the fission yeast [83]. 

How then is the link between replication and recombination established although these events are temporally separated? While Spo11 is responsible for the generation of meiotic DSBs, its interaction with a number of other conserved factors is critical for this function. One of them is Mer2, a pivotal target of both CDK and DDK phosphorylation in the budding yeast [17,18,107]. This modification by both kinases is necessary for DSB formation [18]: Clb5/6-Cdc28 modifies Ser30 of Mer2, and this primes the protein for phosphorylation by Dbf4-Cdc7 on Ser29. Importantly, Dbf4 has been suggested to interact with the replication fork [108], and evidence suggests that the DDK activity that is associated with this machinery phosphorylates Mer2 in replicating regions [109]. Although it remains to be shown whether Mer2 phosphorylation occurs as the replication fork progresses along the DNA, as the direct recruitment of DDK to the traveling replication machinery has not yet been demonstrated, these findings provide a key mechanism for coupling replication with recombination.

Interestingly, while origin activation and DSB formation are separated in time, their joint reliance on CDK and DDK has led to the suggestion that there may be competition for the same kinase activities. Indeed, as described above, the establishment of recombination begins during S phase before breaks are actually formed, and this is mediated through Mer2 phosphorylation by CDK and DDK during pre-meiotic S phase. In light of the quantitative requirements for these kinases during meiosis, it is tempting to speculate that there may be intermediate thresholds of activity that coordinate and ensure the temporal order of replication and recombination.

## 7. Repair of DNA Double-Strand Breaks in Mitotic and Meiotic Cycles

Although the formation of DSBs initiates meiotic recombination, they are among the most deleterious forms of DNA damage and represent a major challenge to genome maintenance. These breaks can have severe consequences, ranging from chromosomal translocations to cell death [110]. Therefore, while meiotic DSBs are programmed events, they also have the potential to threaten genome stability if they are not properly repaired (reviewed in [111]). The preservation of genome integrity requires the function of a number of pathways for the detection and repair of DNA lesions. In this section, we will explore how cells deal with DSBs in mitotic and meiotic cycles as well as the roles of CDK and DDK in these processes.

In proliferating cells, DSBs are repaired via two major mechanisms. In situations where cells have a duplicated genome for use as a template, the preferred pathway is homologous recombination (HR), which takes an identical or similar sequence as a donor. However, when a copy of the genetic information is not available, non-homologous end joining (NHEJ) promotes the ligation of the broken DNA. This occurs through the processing of DNA ends, which may result in nucleotide alterations and thus is generally considered to be more error-prone. Due to the template requirements for these two repair mechanisms, their utilization is directly coupled to cell cycle progression: NHEJ is active throughout the cell cycle but predominant in G1, while HR is restricted to S and G2, when an undamaged template becomes available. This preference has been demonstrated in the budding yeast, where DSBs that are generated in G1 are repaired by NHEJ rather than by HR [112,113]. Moreover, the levels of NHEJ and HR have been shown to be reciprocally regulated throughout the cell cycle in fission yeast: NHEJ is 10-fold higher than HR in G1, while the opposite is true in G2 [114].

Consistent with the quantitative model for cell cycle progression, CDK activity has been demonstrated to be a critical regulator of the choice between these pathways. First, CDK downregulates NHEJ when a donor template is present. For instance, the Xlf1 protein that stimulates DNA end joining undergoes inhibitory phosphorylation by CDK/Cdc2 as fission yeast cells enter G2 [115]. Next, a number of the proteins in the HR pathway are substrates of CDK (reviewed in [6,116,117]). Indeed, CDK/Cdc28 promotes the resection of DSB ends to generate single-stranded DNA overhangs for HR in the budding yeast [112,113]. This requires CDK modification of the Sae2/CtIP endonuclease, as demonstrated in systems ranging from budding yeast to mammalian cells [118,119,120]. The later steps of HR, in which DNA joint molecules that are generated as a result of homology search and strand invasion must be resolved and disentangled, are also dependent on CDK. For example, in the budding yeast, the biochemical activity of the Mms4/Eme1-Mus81 nuclease that is important for joint molecule processing reaches a maximum at G2/M, and this relies on CDK/Cdc28 phosphorylation [121,122,123]. Interestingly, during meiosis, the formation of programmed DSBs occurs in a temporal window following pre-meiotic S phase and prior to chromosome segregation during meiosis I. HR during mitosis and DSB repair during meiosis are related processes, and it has been hypothesized that meiotic recombination is a specialized function that may have evolved from HR [1]. Importantly, CDK substrates in HR during mitotic cycles are similarly crucial for repairing and resolving meiotic DSBs. This includes the Sae2 protein mentioned above, whose phosphorylation is essential for removal of Spo11 from DSB ends and for initiation of meiotic DSB resection [124]. Moreover, the CDK-dependent activity of Mms4-Mus81 promotes the processing of joint molecules prior to chromosome segregation in meiosis I [121]. Finally, DDK activity has also been implicated in the regulation of Mms4-Mus81 in proliferating cells [125]; it is thus possible that this phosphorylation will play a similar role in meiosis. Collectively, the examples described above illustrate the fundamental functions of CDK and perhaps of DDK in the repair of DSBs in both mitotic and meiotic cycles.

## 8. Conclusions

The CDK and DDK kinases are essential regulators of genome duplication and maintenance in proliferating cells and during meiosis. Many of their roles in mitotic cycles have correlates in sexual reproduction, but cells have also implemented meiosis-specific adaptations of their modulation and functions, some of which have been presented in this review. Intriguingly, despite the complexity of the control of these kinases, orderly progression through meiosis may simply rely on the levels of CDK and DDK activities, as is the case in mitotic cycles. Since meiosis involves a number of events that do not normally occur in vegetatively growing cells, the higher activities required for later meiotic stages may provide a greater dynamic range that allows for additional intermediate thresholds to ensure the proper succession of non-overlapping processes. Therefore, the precise profiles of CDK and DDK activities may be critical both to drive and temporally orchestrate the diverse steps in gametogenesis.
